# The impact of moderate-intensity swimming exercise on learning and memory in aged rats: The role of Sirtuin-1

**DOI:** 10.22038/IJBMS.2021.58145.12920

**Published:** 2021-10

**Authors:** Ulker Tunca, Mustafa Saygin, Ozlem Ozmen, Rahime Aslankoc, Arzu Yalcin

**Affiliations:** 1 Suleyman Demirel University, Faculty of Medicine, Department of Physiology, Isparta, Turkey; 2 Department of Pathology, Faculty of Veterinary Medicine, Burdur Mehmet Akif Ersoy University, Burdur, Turkey

**Keywords:** BDNF, CREB, Learning-memory, Sirtuin-1, Swimming exercise

## Abstract

**Objective(s):**

The purpose of this study was to evaluate the effect of moderate-intensity swimming exercise on learning and memory by the Morris water maze test. Changes in the expressions of cyclic AMP-response element-binding protein (CREB) and brain-derived neurotrophic factor (BDNF) proteins alternative pathway which were activated by sirtuin-1 (SIRT-1) were investigated.

**Materials and Methods:**

The study included thirty-two male Sprague-Dawley rats (350-500 g, 11-12 and 15–16 months old). The rats were randomly divided into four groups with 8 rats in each group. The groups were designed as follows: Control-1 (11-12 months), Exercise-1 (11-12 months), Control-2 (15-16 months), Exercise-2 (15-16 months). Moderate-intensity exercise was assigned for 30 min/day, 5 days/week, for the whole training period of 8 weeks.

**Results:**

There were statistically significant differences between the groups on the third day (*P=*0.005) when swim speeds increased in the exercise groups. There was a statistically significant difference between Exercise 1 and Exercise 2 groups, the entries in the platform zone decreased in Exercise 2 group (*P=*0.026). While there were no histopathological findings observed in any group, increased SIRT-1, BNDF, and CREB expressions were seen in exercise groups compared with control groups.

**Conclusion:**

In aged rats exercising at moderate intensity, increased expression of CREB and BDNF, and SIRT-1 could improve hippocampal-dependent memory.

## Introduction

Dementia has a serious impact on age-related morbidities in today’s elderly population (1, 2). Exercise affects multiple brain functions by creating new synaptic structures, activating the mechanisms of brain plasticity, enhancing neurogenesis and vascularization. Exercise increases brain metabolic capacity and provides some positive effects on the anti-oxidant defense system (3, 4).

Homeostatic balance occurs between free radicals and anti-oxidants. Deterioration of the homeostatic balance in favor of oxidants is known as oxidative stress. Even though high-intensity exercise leads to increased oxidative stress, a similar exercise performed in low-intensity provides an increase in endogenous anti-oxidants with respect to the hormesis theory (5). As a result, exercise itself functions as an anti-oxidant because it enhances the expression of anti-oxidant enzymes (6). The effect of resistant aerobic training on the improvement of cognitive capacity is achieved by increasing the level of neurotrophic factors that increase neuronal survival, synaptic development, and plasticity in rats (7-9). 

It is well known that different exercise programs increase neuroplastic activity through the brain-derived neurotrophic factor (BDNF)-Tropomyosin-related kinase B (TrkB) signaling that enhances neurogenesis. BDNF is associated with neural development and functions such as new neuron formation, dendritic growth, and long-term potentiation in neurons. The best result in the dose-dependent relationship between the duration and intensity of exercise and BDNF has been reported to be linked to regular moderate exercise (10-12). Exercise regulates the activity of transcriptional regulatory factors such as cAMP-response element-binding protein (CREB), which is very important for learning and memory. In addition, a significant rise in sirtuin-1 (SIRT-1)/mRNA signaling related to synaptic plasticity and hippocampal memory was reported to be associated with physical activity (13).

The sirtuin family is in the class III Histone Deacetylases group, which has nicotinamide adenine dinucleotide (NAD^+^)-dependent histone deacetylase. It has been shown that a decrease in SIRT-1 with age may decrease the levels of BDNF, an important neurotrophic factor that regulates learning, memory, and synaptic function in the brain (14, 15).

Although the relationship between exercise and memory is proven, which paths are involved in the development process of the brain, and the way they are activated still need to be discovered. Moreover, molecules linking SIRT-1 to learning and memory have not yet been thoroughly investigated. The purpose of our study was to evaluate the learning and memory effectiveness in rats after the application of swimming training by the Morris water maze test, and then, to investigate whether CREB and BDNF proteins are an alternative signaling pathway associated with SIRT-1 activation.

## Materials and Methods

***Ethical approval***

This study was conducted at the Research Laboratory of Suleyman Demirel University, Faculty of Medicine, Department of Physiology. All procedures were certified and approved by the Animal Experiments Local Ethics Committee of Mehmet Akif Ersoy University, Burdur, Turkey (19.07.2018/377).

***Animals***

In this study, a total of thirty-two male Sprague Dawley rats, weighing 350-500 g were used. Rats were kept at a constant temperature of 24-26 ^°^C, 55-60% humidity, 12/12 hr light/dark cycle. Food (Standard rat chow, Korkuteli Yem, Turkey) and water were given *ad libitum*. The cages were changed weekly, and rats’ fluids were changed every two days.

***Experimental design***

The rats were randomly divided into 4 groups with 8 rats in each: Control - C1 (11-12 months old), Exercise-E1 (11-12 months old), Control-C2 (15-16 months old), Exercise-E2 (15-16 months old). The swimming program consisted of two stages: adaptation and training. The rats were floated by keeping the training time constant for 30 min. Moderate exercise intensity was applied at an intensity of 30 min/day, 5 days/week, with the training period lasting 8 weeks. After the 8-week exercise program, the learning-memory tests of the rats were performed using the Morris water maze test. Learning experiments were carried out for at least 20 trials for each animal, 5 times a day after 8 weeks, for 4 days. Swimming exercises and learning-memory analyzes were performed between 09:00 am and 12:00 pm.

***Exercise program***

Daily swimming training was performed at the water temperature of 32±2 ^°^C, kept constant by a thermostat immersed in a wide water tank [130 cm diameter×45 cm height]. Care was taken that the rats never touched the ground with their feet during the swimming exercise**.** The animals were swimming in groups of eight. The swimming program consisted of two stages: adaptation and training. Adaptation training time was gradually increased from 10 min in the first week to 30 min on the last day (16). The adaptation stage was used to prevent water-induced stress-causing excessive physiological changes. Following one week of the adaptation period, the actual exercise training started in proportion to the adaptation period. The intensive training period lasted 30 min/day, 5 days/week for 8 weeks (17, 18). In the training period, moderate-intensity exercise was assigned (19) at the same time each day (from 09:00 am to 11:00 am). After swimming training, the rats were dried with a towel and remained warm under an electric heater.

***Morris water maze (MWM) procedures***

After 8 weeks of the exercise program, rats’ learning and memory abilities were evaluated using the Morris water maze test. A round galvanized water tank (pool) (130 cm diameter and 45 cm deep) was used for learning experiments. The water maze was filled with water and heated to 23 ^°^C before the experiment. The water was made opaque by adding non-toxic paint. During the learning trials, the animals did not see the hidden platform under the water. The pool was divided into four quadrants. Quadrants were ordered from 1 to 4 and the target quadrant was determined as the 2nd quadrant. In the 2^nd^ quadrant, a hidden platform was put 2 cm below the water level. In this way, the rats were expected to find the hidden platform. Each rat was allowed to explore the platform for 1 min. If a rat did not find the platform, it was put on the platform manually and was allowed to explore the space for 30 sec. For the rats that found the platform, a 30 sec rest period was given on the platform to ensure that they had learned to locate the platform (20). After the period of 8 weeks, learning experiments were carried out 5 times a day, at least 20 trials per animal, for 4 days (21). On the 5^th^ day of the 9^th^ week, all groups were tested for memory. The memory test was performed by recording the time spent by each rat in the destination quadrant and repeated for every group of animals. Daily data for animals in all groups were evaluated separately within the groups and between the groups (22, 23). 

All swimming training was recorded and transferred to a computer by a Sony camcorder SSC-G118 (Sony, Tokyo, Japan) placed on the ceiling in the center of the pool. Data were examined using the Smart v3.0 application. The latency to the target, distance to target, mean directionality, entries in the platform zone, number of zone crossings, the time spent on the destination quadrant, and mean swim speed were evaluated as markers of learning. 

***Biochemical analysis***

*Oxidative stress parameters*

Hippocampus samples were homogenized, and Total Anti-oxidant Status (TAS) and Total Oxidant Status (TOS) levels were evaluated by the automated colorimetric measurement method developed by Erel. In hippocampus tissue homogenates, TAS and TOS levels were measured by a Rel Assay Commercial Kit (Rel Assay Diagnostics, Turkey). The results were presented as mmol Trolox equiv./lt (24) and μmol H_2_O_2_ equiv./lt (25). The Oxidative Stress Index (OSI) was calculated as the ratio of TOS to TAS. The mmol value in the TAS test unit was converted to µmol units as in the TOS test, and OSI was calculated; dividing the ratio of TOS (μmol H_2_O_2_ equiv./lt) to the result of the product of TAS (mmol Trolox equiv./lt) multiplied by 10.

***Histopathological analysis***

The hippocampus samples from the rats were fixed in 10% buffered formaldehyde solution during the necropsy. After two-day fixation, tissues were trimmed and placed into the tissue processing cassettes. They were left in formalin one more day, then placed on the tissue tracking device (Leica ASP300S) and routinely processed and embedded into the paraffin wax. After 4-5 hr of cooling of the blocks the Leica 2155 rotary microtome serial sections of 5 microns thick to prepare normal and polylysined coated slides. The sections were then stained with hematoxylin and eosin (HE) and examined microscopically. Histopathological changes were graded in a blind manner and lesions were scored to evaluate the pathological findings. Scores were made related to hyperemia, hemorrhages, edema, and vacuolar degeneration.

***Immunohistochemical analysis***

The streptavidin-biotin method was applied to 3 separate serial sections taken on to polylysined slides for the immunoperoxidase staining. The sections were stained with Sirtuin-1 [Anti-SIRT-1 antibody [E104] (ab32441), Abcam, 1/100 dilution], BDNF [Anti-BDNF Picoband antibody (PB9075), BosterBio, 1/100 dilution] and CREB [Anti-CREB/CREB1 Picoband antibody (PB9100), BosterBio, 1/100 dilution] according to the immunohistochemical procedure to determine the reaction. The stained tissues were examined under a light microscope (Olympus CX41). The same procedure was repeated without the primary antibody for negative controls. Microphotography and morphometric analysis were conducted using the Database Manual CellSens Life Science Imaging Software (Olympus Corporation, Tokyo, Japan). To determine the percentage of positive cells, a total of 100 cells consisting of 20 cells from 5 separate regions were counted under a 40X objective. The percentage of immunostained cells in each sample was calculated and statistical analysis was performed.

***Statistical analysis***

Statistical evaluations were made using SPSS 22.0 for Windows. Descriptive statistical analysis was used to present the data of the Morris water maze test. Statistical analysis was conducted by Student’s t-test and Repeated measures analysis of variance (ANOVA). Kolmogorov-Smirnov test was performed to test for normal distribution, and a non-parametric Mann-Whitney U test was used for intergroup comparisons of oxidative stress parameters. A *P*-value of<0.05 was considered statistically significant. 

## Results

***MWM test results***

*Learning period data*

The latency to the target: diurnal intragroup comparisons indicated that MWM performance was markedly decreased on days 2 and 4 when compared with the performance on day 1 (*P*=0.001) (Table 1). The number of zone crossing: the number is remarkably decreased on days 2 and 4 compared with the data on day 1 (*P*=0.001). Distance to target: the distance significantly decreased on days 3 and 4 compared with the data on day 1 (*P*=0.001). Mean directionality: is significantly increased on Days 1 and 3 compared with the data on day 1 (*P*=0.001). Entries in the platform zone: intragroup comparisons revealed significant differences (*P*=0.005). There was a statistically significant difference between Control 1 and Exercise 1 groups, and the number of entries in the platform zone was increased in Exercise 1 group compared with Control 1 group (*P*=0.022). There was a statistically significant difference between Exercise 1 and Exercise 2 groups in terms of the number of entries in the platform zone, which was decreased in the Exercise 2 group (*P*=0.026).

*Memory Period*

Time spent on the destination quadrant. Analysis of the data from the trial test of the time spent on the destination quadrant using repeated measures analysis demonstrated no significant differences between the groups (*P*>0.05); though, the time spent on the destination quadrant increased in the C-2 group compared with the E-2 group on Day 4 (*P*=0.048). In terms of memory, there was a significant difference between C-1 and E-1 groups in terms of the time spent on the destination quadrant at the trial test. An increase in the average swimming speed in exercise groups is another parameter that supported memory development (Table 2).

***Mean swim speed***

Mean swim speeds were determined by measuring path length (cm) and escape latency (sec) that each rat spent locating the hidden platform. When comparing the data, we found statistically significant differences between the groups on day 3 (*P*=0.005), and swim speeds increased in the exercise groups.

***Biochemical results***

*Oxidative stress parameters*

The difference between TAS and TOS levels of none of the groups was statistically significant. There was a statistically significant difference only between Exercise 2 and Control 2 groups in terms of hippocampal OSI markers (*P*=0.037 ) (Table 3).

***Histopathological results***

**Figure 1:**
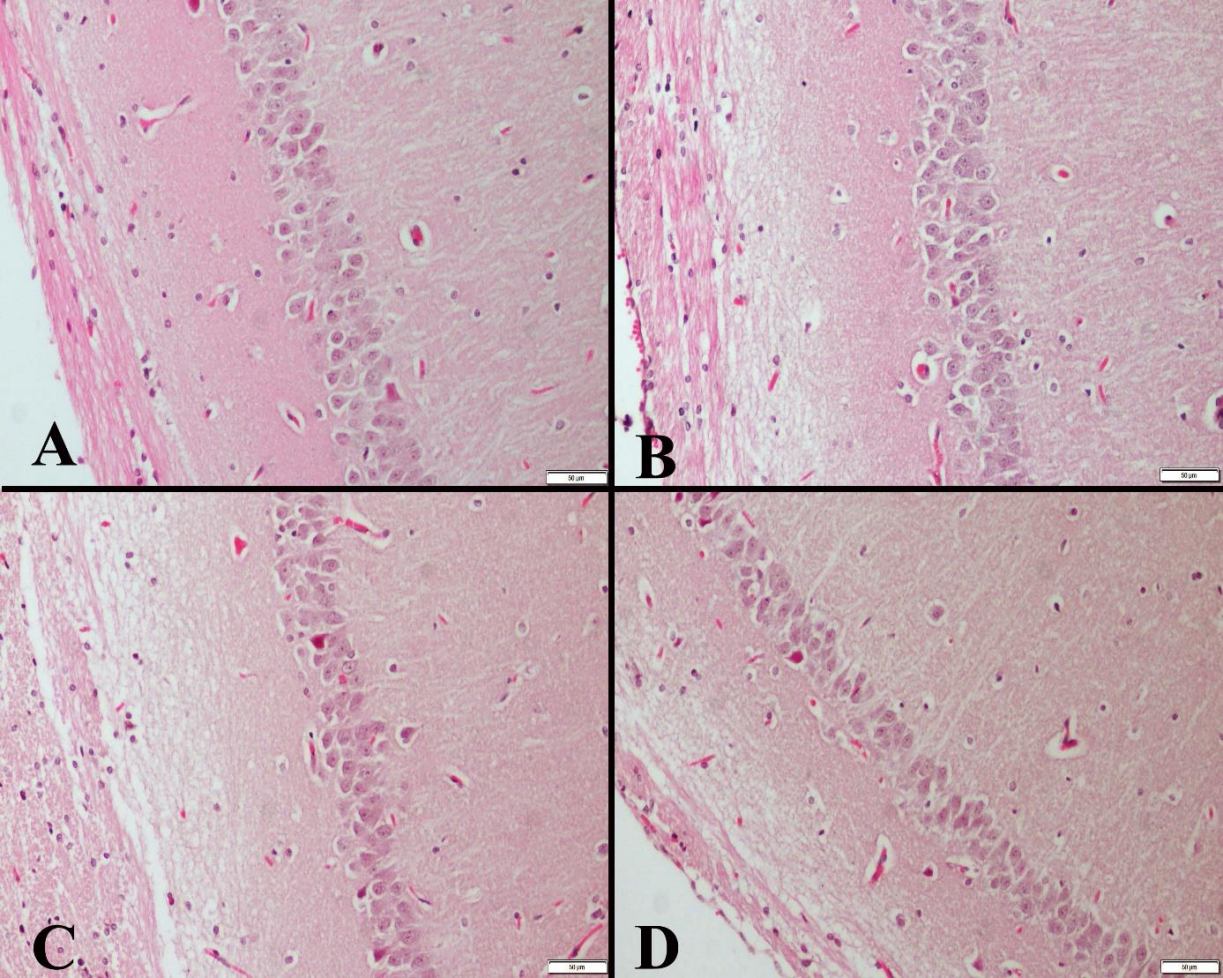
Histopathological appearance of the hippocampus between the groups of rats

 During the processing of the brain tissues, maximum attention was paid to complete the procedures without damaging the tissue. At the histopathological examination of the hippocampuses, no significant finding was observed in any group. On the other hand, hyperemia was observed in all groups (Figure 1).

***Immunohistochemical results***

**Figure 2:**
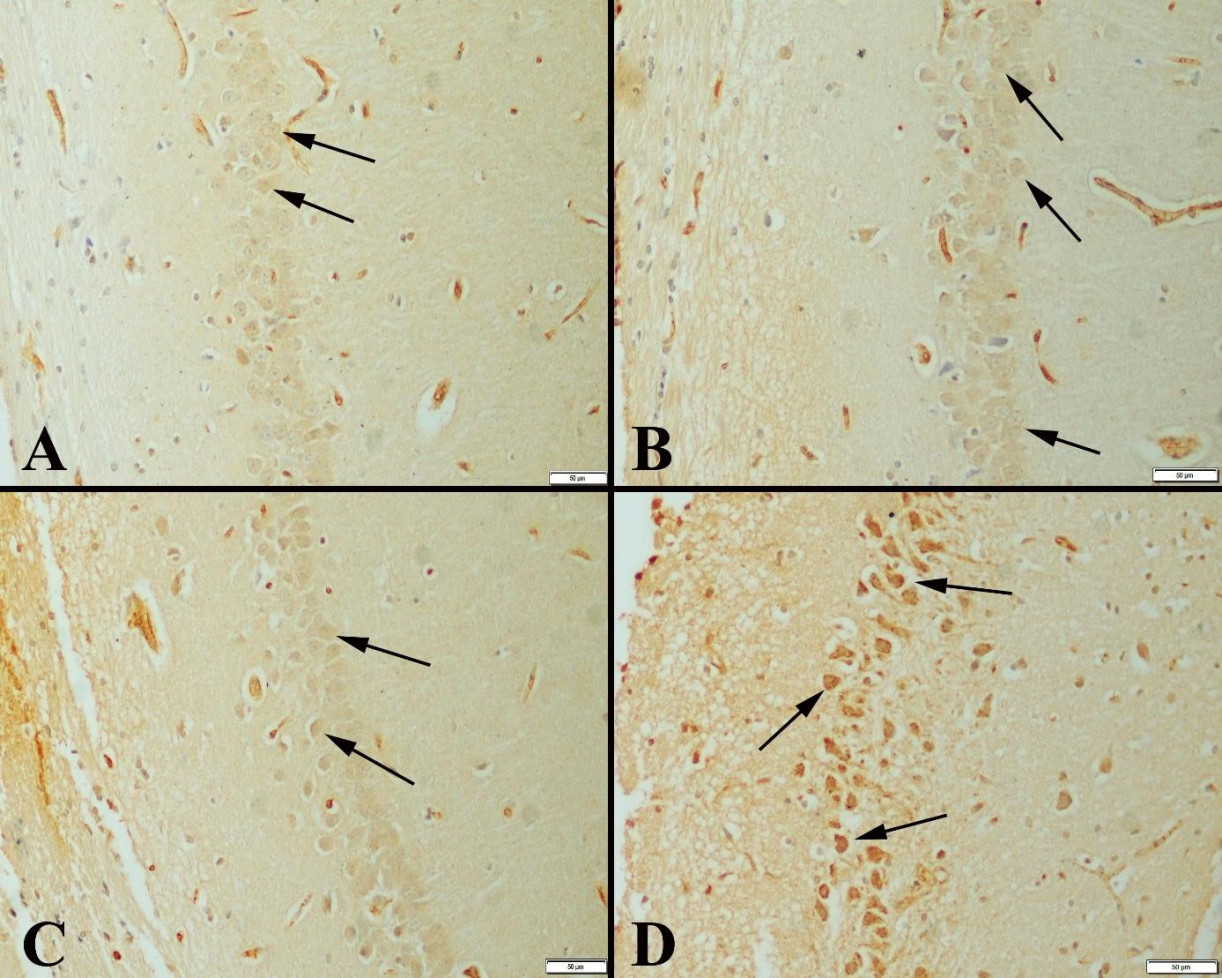
CREB expressions in hippocampus between the groups

**Figure 3:**
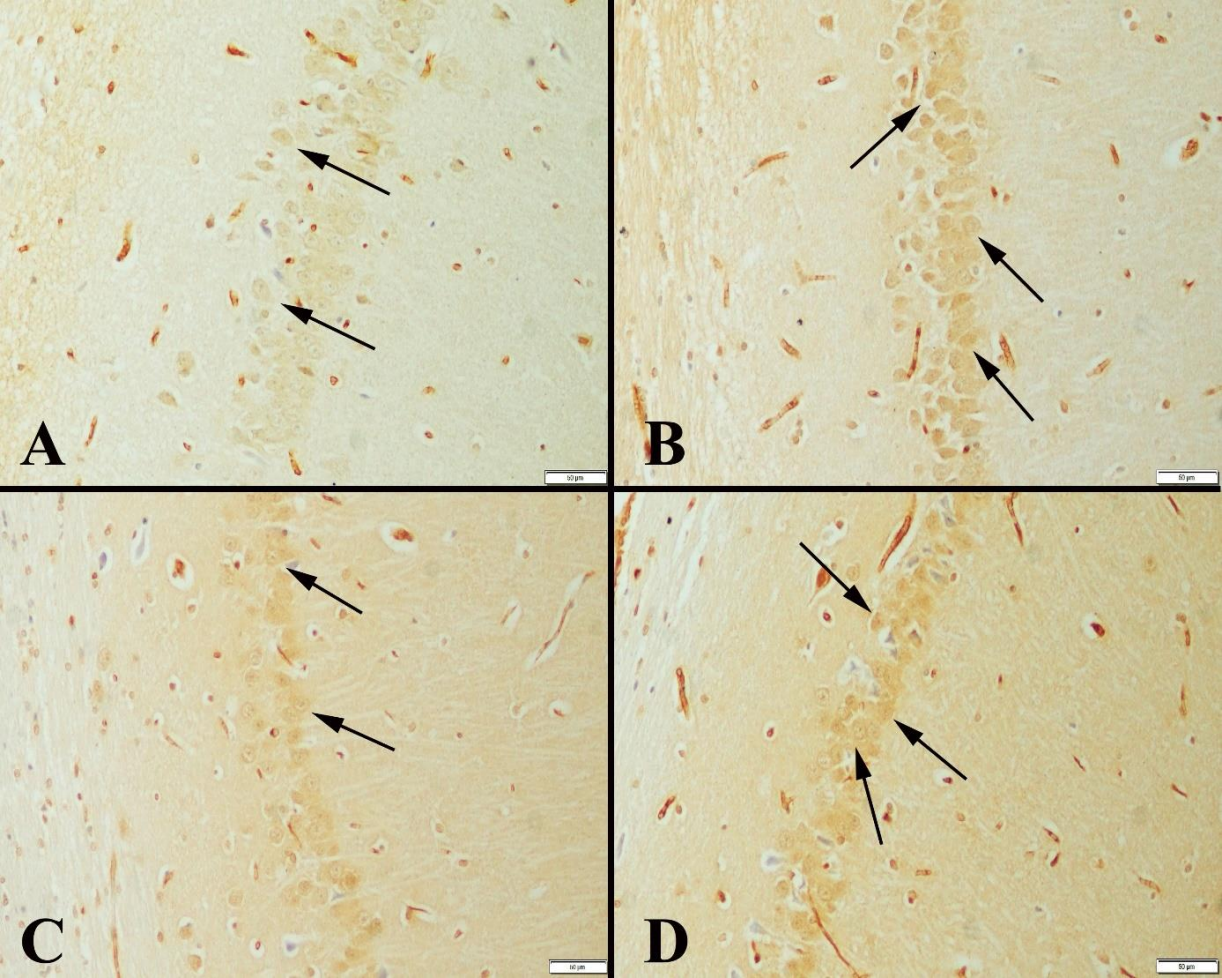
BDNF immunoexpressions in hippocampus between the groups of rats

**Figure 4:**
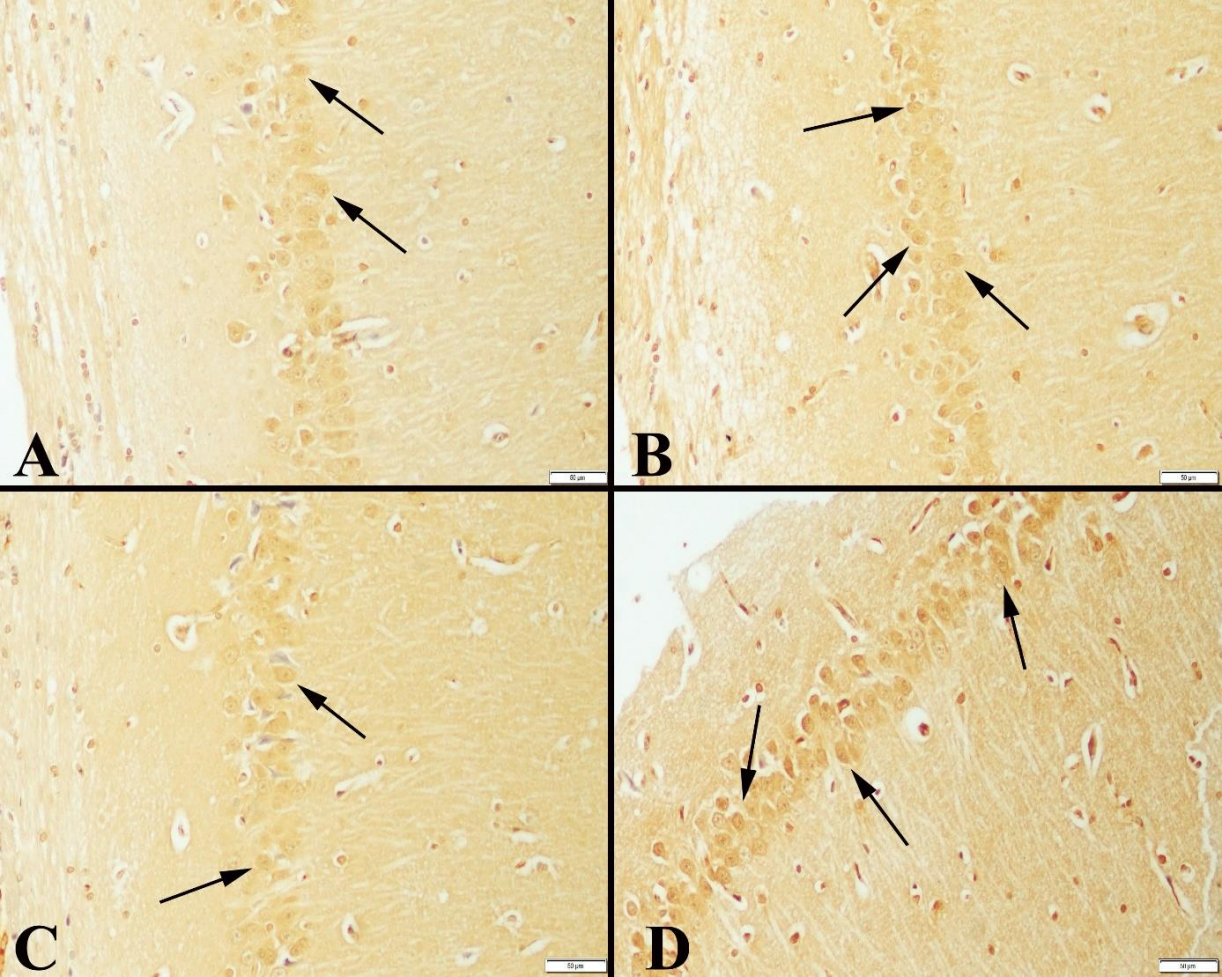
SIRT1 expressions in hippocampus among the groups of rats

The immunohistochemical examination of the hippocampus marked an increase in expressions of CREB, BDNF, and SIRT-1 in neurons in exercise groups compared with the control groups. In addition, expressions increased the duration of the study. CREB immunohistochemical examination of the hippocampuses showed immunopositive reaction in the neurons. It was observed that the expression was more marked especially in the exercise groups and there was a positive expression in a much larger number of neurons (Figure 2). BDNF immunoreactions were examined among the groups, and a significant increase was observed in exercise groups compared with the control groups. The positive immunoreaction was detected homogeneously and in the cytoplasm (Figure 3). Immunohistochemical findings of the hippocampus were also observed in SIRT-1 activity groups and increased immunoreactions were observed in exercise groups (Figure 4). Statistical analysis of the number of positive cells in terms of all markers examined among the groups is given in Table 4.

**Table 1:** The latency to the target as a groups

**Groups**	**TAS ** **(mmol Troloxequivalents/L)**		**TOS ** **(mmol H**_2_ **O**_2_ ** Equiv./L)**		**OSI**	
	Mean±SD	*P*-value	Mean±SD	*P*-value	Mean±SD	*P*-value
**Control 1**	0.70±0.18	NS	4.87±0.61	NS	7.36±2.13	NS
**Exercise 1**	1.13±0.75	NS	5.89±2.75	NS	5.66±0.82	NS
**Control 2**	1.09±0.36	NS	5.58±1.04	NS	5.37±0.93	NS
**Exercise 2**	1.13±1.23	NS	6.26±4.526	NS	9.62±8.55^a^	a:0.037

Values are presented as mean±SD. Relationships between groups and results of learning peirod data were evaluated by independent t test

^a^ *P<*0.05 versus control group

**Table 2:** The time spent on the destination quadrant as a groups

**Group**	**SIRT-1**	**BDNF**	**CREB**
Control -1 (C-1)	10.50±1.04	7.16±1.47	12.00±2.28
Exercise -1 (E-1)	17.66±0.81	16.00±1.26	21.33±2.16
Control -2 (C-2)	14.33±1.21	7.00±1.54	9.50±1.04
Exercise -2 (E-2)	16.50±1.64	11.33±1.21	17.16±1.94
***P*** **-value**	C-1 and E-1 <0.001 C-1 and C-2 <0.001 C-1 and E-2 <0.001 E-1 and C-2 >0.05 E-1 and E-2 <0.01 C-2 and E-2 >0.05	C-1 and E-1 <0.001 C-1 and C-2 >0.05 C-1 and E-2 <0.001 E-1 and C-2 <0.001 E-1 and E-2 <0.001 C-2 and E-2 <0.001	C-1 and E-1 <0.001 C-1 and C-2 >0.05 C-1 and E-2 <0.001 E-1 and C-2 <0.001 E-1 and E-2 <0.01 C-2 and E-2 <0.001

Values are presented as mean ± SD. Relationships between groups and results of learning peirod data were evaluated by independent t test

^a^ *P<*0.05 versus control group

**Table 3:** Oxidative stress markers of hippocampus tissue as a groups

Groups	TAS (mmol Troloxequivalents/l)		TOS (mmol H2O2 Equiv./l)		OSI	
	Mean±SD	*P*-value	Mean±SD	*P*-value	Mean±SD	*P*-value
Control 1	0.70±0.18	NS	4.87±0.61	NS	7.36±2.13	NS
Exercise 1	1.13±0.75	NS	5.89±2.75	NS	5.66±0.82	NS
Control 2	1.09±0.36	NS	5.58±1.04	NS	5.37±0.93	NS
Exercise 2	1.13±1.23	NS	6.26±4.526	NS	9.62±8.55^a^	a:0.037

Values were expressed as means±SD. The relationships between groups were evaluated by Bonnferroni one-way ANOVA

^a ^Compared with Control 2

NS: Not siginificant; TAS: Total anti-oxidative status; TOS: Total oxidative status; OSI: Oxidative stres index

(A) C-1, (B) E-1, (C) C-2 and (D) E-2 groups. No significant difference was found in any the group at the histopathological evaluation. Only hyperemia was observed similarly in all groups

HE, scale bar=50 µm

(A) Slight immunoreactions in neurons (arrows) in group C-1 (B) Increased immunoreactions (arrows) in E-1 group (C) Increased immune-positive reaction in some neurons (arrows) in group C-2 (D) Marked increased immunoexpressions (arrows) in E-2 group. Streptavidin biotin peroxidase method, Scale bars = 50 µm

(A) Slight immunoreactions in some neurons (arrows) in the C-1 group (B) Significantly increased expressions (arrows) in the E-1 group. (C) Increased immunopositive reactions (arrows) in some neurons in the C-2 group (D) Marked increase in immunoreactions (arrows) in many neurons in the E-2 group. Streptavidin biotin peroxidase method, scale bars-50 µm

**Table 4:** SIRT-1, BDNF and CREB statistical analysis of immunohistochemical positive cell numbers of hippocampus of rats between the groups

Groups	SIRT-1	BDNF	CREB
Control -1 (C-1)	10.50±1.04	7.16±1.47	12.00±2.28
Exercise -1 (E-1)	17.66±0.81	16.00±1.26	21.33±2.16
Control -2 (C-2)	14.33±1.21	7.00±1.54	9.50±1.04
Exercise -2 (E-2)	16.50±1.64	11.33±1.21	17.16±1.94
*P value *	C-1 and E-1 <0.001 C-1 and C-2 <0.001 C-1 and E-2 <0.001 E-1 and C-2 >0.05 E-1 and E-2 <0.01 C-2 and E-2 >0.05	C-1 and E-1 <0.001 C-1 and C-2 >0.05 C-1 and E-2 <0.001 E-1 and C-2 <0.001 E-1 and E-2 <0.001 C-2 and E-2 <0.001	C-1 and E-1 <0.001 C-1 and C-2 >0.05 C-1 and E-2 <0.001 E-1 and C-2 <0.001 E-1 and E-2 <0.01 C-2 and E-2 <0.001

Values were expressed as means± SD. The relationships between groups were evaluated by Bonnferroni one-way ANOVA

SIRT-1: Sirtuin-1, BDNF: Brain Derived Neurotrophic Factor, CREB: cyclic amp-response element binding protein

(A) Slight immunoreactions in some neurons (arrows) in the C-1 group (B) Moderate increase in immunoreactions (arrows) in the E-1 group. (C) Mild immunopositive reuse in some neurons in the C-2 group (D) Marked increase in immunoreactions in many neurons (arrows) in the E-2 group. Streptavidin biotin peroxidase method, scale bars=50 µm

## Discussion

Looking at the clear relationship between learning and memory improvement and continuous regular exercise, there is no doubt that it can reverse the cognitive decline with aging (26, 27). The development of learning-memory function with the Morris water maze test confirmed that continuous regular moderate-intensity exercise made it more effective (28, 29). 

We can say that regular moderate-intensity swimming exercise may be beneficial in old age. Statistically significant differences were found between the groups in terms of the latency to the target, number of zone crossings, distance to target, mean directionality, and entries in the platform zone in MWM data which proved the effect of exercise on learning development.

Van Praag *et al*. reported that swimming exercise could not reach any meaningful results in the comparison of neuronal cell proliferation and the Morris water maze test (30).

However, studies with different exercise doses speak of plasticity effectiveness. a research suggested that an 8-week moderate exercise could be effective in protecting against learning-memory and neurodegeneration and could improve plasticity by directly affecting synaptic structure (21). Long-term studies on moderate exercise training suggest that aging can be used as a strategic method to delay and cure memory loss (26, 27, 29).

Van Praag *et al*. reported a significant difference in the length of the swim path and the time of finding the platform. The exercise group was faster in the swim speed compared with other groups (30). Similarly, in our study, it was found that the swim speed increased in the exercise groups. A statistically significant difference was found between latency of target, zone transition number, distance to target, mean directionality to target, and entries in zone platform tasks in Morris data, which proved the effectiveness of exercise in learning development. Exercise has positive effects on learning, but more evidence is needed to define age-related exercise intensity.

During the aging period, physical activity reduces the risk of neurocognitive impairment and dementia (31). The relationship between dementia from old age and exercise is a long-standing issue. The results showed that both oxidative stress reduction and increased expression of neurotrophic factors in the hippocampus may have positive effects on memory performance in elderly rats (32). A decrease in oxidative stress markers was observed in the brain tissue in the exercise group compared with the control group (33). In general, it is seen in the literature that regular moderate aerobic exercise increases anti-oxidant capacity in the brain (34). In our data, there was a significant change in OSI findings, although it was proportional to the above. We cannot say the same about TAS and TOS findings. We think that the limitation of the study may be the small number of elderly rats.

Baek and Kim treadmill exercise studies emphasize microglial activity and may be protective against apoptosis. They reported that exercise training against defects in the brain in the aging process with dementia showed decreased Bax expression and increased Bcl-2 expression (35). In our study, hyperaemic centers were found in histopathological findings. In our literature scans, we could not find compatible data on this subject. We hypothesize that the source of hyperemia may be a physiopathological process that may be caused by aging. In addition, if exercise is a stress factor, it can be said that the rats in the study groups are elderly.

A study reported that swimming exercises that activate the Akt and CREB signaling pathways in the hippocampus could increase short-term memory in the study of elderly mice (36). Research emphasized that high-intensity aerobic exercise may affect memory function adversely in mice by negatively affecting CREB and ERK1 / 2 pathways in the neuroplasticity of the brain structure (37). Vaynman *et al*. showed that CREB expression increased significantly immediately after exercise (38). Immunohistological findings in our study showed that CREB increased significantly in the hippocampal tissue of moderate swim exercise groups compared with controls. There was also a significant difference in the relationship between the two age groups (E1-E2). When we look at the effect of age, less expression in exercise group 2 was explained by the lesser CREB expression due to neurodegenerative processes progressing with age. 

A study reported that increased expression of BDNF could increase synaptic conduction and hippocampal LTP activity (39). Research showed BDNF is associated with learning-memory, especially in long-term empowerment, and may increase its effectiveness due to trial frequency (40). Concerned with the relationship between exercise dose and BNDF expression, a study reported that low-intensity exercise did not increase BDNF expression in hippocampus tissue of rats (41). Immunohistological findings in our study showed that BDNF increased in hippocampal tissue of exercise groups. It was also revealed that exercise could be effective in decreasing the decline caused by aging between exercise 1 and exercise 2 groups. The results we found were consistent with previous studies showing that BDNF-mediated learning-memory function was developed according to the above data (42-44). 

It has been found that exercise stimulates synaptic plasticity markers in the hippocampus via a BDNF-mediated mechanism and increases synapse I level with calcium and CREB protein. The association between exercise-induced cognitive improvement and BDNF is known. The inhibition of BDNF expression indicated the inhibition of the ability to increase CREB and Synapsin I from the downstream signal pathways under the control of exercise-induced BDNF. In particular, the association of BDNF and CREB-mRNA expression with learning memory was found (45-47).

Molecular mechanisms regulating hippocampal plasticity have been predicted as SIRT1/microRNA, CREB/BDNF, and AKT/GSK-3β signaling pathways. SIRT1 is found in all areas of the brain, particularly in the hippocampus, prefrontal cortex, and basal ganglia. These results suggest that SIRT1 modulates synaptic plasticity and memory formation in the hippocampus of mice. SIRT1 has been shown to play a role in regulating memory and plasticity through the CREB/BDNF pathway in the hippocampus (48). Our study showed that SIRT1 expression increased in the exercise groups compared with the control groups. These data, in line with the literature, revealed that regular exercise may have an effect on the increase in SIRT1 expression during the aging process.

## Conclusion

According to our results, exercise increased SIRT1, BDNF, and CREB expression in rats in the aging process. Our findings suggest that exercise training could affect the risk factors that cause cognitive failure by reducing cell death in hippocampal cells. In addition, these research data showed results that supported our opinion that BDNF and CREB expression were activated in parallel with the increase in SIRT1. SIRT-1, BDNF, and CREB pathways may be an alternative pathway for increasing synaptic plasticity in aging rats. The presence of this pathway needs to be addressed precisely in the inter-cellular signal and protein assemblies under deacetylation. Our findings should be supported by further studies

## Authors’ Contributions

All the authors participated in the design and interpretation of the study, data analysis, and the review of the manuscript. UT and AY conducted the experiment and collected the data, RA and MS were responsible for the data analysis. OO performed histopathological and immunochemical analyses and provided methodological and technical guidance. UT wrote the manuscript, MS and RA reviewed the manuscript. All the authors read and approved the final manuscript. 

## Conflicts of Interest

The authors declare that there are no conflicts of interest.
